# Acute Visual Disturbances and Ataxia Secondary to Attempted COVID-19 Prophylaxis With Ivermectin in a Nine-Year-Old

**DOI:** 10.7759/cureus.35944

**Published:** 2023-03-09

**Authors:** Pranshu Bhardwaj, Daniel Valladares, Colleen K Gutman, Judith K Lucas, Maria N Kelly

**Affiliations:** 1 Department of Pediatrics, University of Florida College of Medicine, Gainesville, USA; 2 Department of Emergency Medicine, University of Florida College of Medicine, Gainesville, USA; 3 Department of Pediatrics, Division of Emergency Medicine, University of Florida College of Medicine, Gainesville, USA

**Keywords:** child maltreatment, drug toxicities, neurotoxicity syndromes, covid-19, ivermectin

## Abstract

Ivermectin is an antiparasitic agent listed as an essential medication by the World Health Organization. Ivermectin utilization has increased due to the popular, though inaccurate, perception of its use in COVID-19 management. Poison Control Central calls regarding ivermectin toxicity have increased 245% since pre-pandemic baselines. This case study illustrates the clinical presentation of ivermectin toxicity in a nine-year-old child with acute vision changes and ataxia. The child was given 60 mg (1 mg/kg) of veterinary-grade ivermectin by a parent, 10 times the clinically recommended dose of 0.1 mg/kg, as prophylaxis after household exposure to COVID-19. Ten hours later, the child developed new-onset blurry vision, a perception of red dots in the peripheral vision, dizziness, and balance issues. Physical examination was notable for pulsating pupils, ataxia, and dysmetria. Symptoms resolved completely after 10 hours. Ivermectin ingestion is an important diagnostic consideration in children presenting with similar symptoms. We hope our case aids in the identification of ivermectin toxicity and hastens necessary supportive measures.

## Introduction

Ivermectin is an antiparasitic agent listed as an essential medication by the World Health Organization [1]. It is primarily used for the treatment of scabies, Strongyloides stercoralis, and Onchocerca volvulus. Ivermectin has recently gained popularity as a prophylactic agent against severe COVID-19 infections despite strong evidence against its efficacy [2]. Reis and colleagues completed a double-blind, randomized, placebo-controlled trial with 1,358 patients presenting with COVID-19 symptoms for seven days and at least one risk factor for disease progression. Patients were randomized to either receive 400 µg/kg of body weight ivermectin once daily for three days or a placebo, with clinical endpoints being hospitalization or an emergency room visit due to worsening COVID-19 symptoms. They found no significant or clinically meaningful lower risk of medical admission or a prolonged emergency department observation with ivermectin [2]. Naggie and colleagues completed a similar trial with 1,206 adult participants and a dose of ivermectin increased to 600 µg/kg of body weight for six days and found that the median time to sustained recovery was 11 days in both groups [3].

Multiple large randomized clinical trials have demonstrated that ivermectin is not effective for prophylaxis against, nor treatment of, COVID-19 [2,3]. However, inaccurate interpretations of data related to ivermectin use in COVID-19 led to the global propagation of misinformation [4,5]. Despite widespread evidence against its efficacy, ivermectin continues to be sought out, often through informal channels. Individuals may obtain ivermectin without a prescription. As a result, the Poison Control Central calls regarding ivermectin toxicity have increased by 245% since pre-pandemic baselines [6]. There are few descriptions of oral ivermectin toxicity in children. We describe our experience caring for a child with acute ivermectin toxicity secondary to attempted COVID-19 prophylaxis.

This article was previously presented as a poster presentation at the 2022 American Academy of Pediatrics National Conference and Exhibition held in Anaheim, California, on October 9th, 2022.

## Case presentation

A previously healthy nine-year-old was transferred from a free-standing emergency department (ED) with acute vision changes and ataxia. Twelve hours prior to presentation to the referring ED, the patient’s parent administered 60 mg (1 mg/kg) of veterinary-grade ivermectin. The ivermectin formulation was intended for livestock such as bovines and was concentrated at 10 mg/mL. The therapeutic dose for a parasitic infection is typically 0.1 mg/kg; the administered dose was 10 times this amount. The parent administered ivermectin to prevent COVID-19 infection after household exposure. Ten hours after medication ingestion, the patient developed new-onset blurry vision, the perception of red dots in the peripheral vision, dizziness, and imbalance. At presentation, the patient had no vomiting, nausea, weakness, fever, shortness of breath, dysuria, or headache. The child had no past medical history of vision difficulties, recent febrile illness, or recent head trauma. The child did have a history of obstructive sleep apnea and asthma, which was well-controlled with albuterol. The rest of the child’s medical history, family history, and surgical history was non-contributory. The child resided with their parents in a rural setting, attended and was doing well in school, and had no pet or tobacco exposure. The child’s vaccinations were up to date. The child had previously received ivermectin from their parents four months earlier for COVID-19 prophylaxis without developing similar symptoms. At that time, the veterinary-grade ivermectin was meant for small pets such as dogs and was concentrated at 1 mg/mL.

On arrival at our pediatric ED, the child’s physical examination demonstrated a blood pressure of 112/63 mmHg, temperature of 37°C, and respiratory rate of 20 breaths per minute, all of which were age-appropriate. The child was saturating 99% on room air and was noted to have a body mass index of 15.22 kg/m^2^, which is the 28th percentile for their age. The patient was well-appearing, did not appear malnourished, and was not in any acute distress or diaphoretic. Head and neck examination was positive for moist mucous membranes and negative for evidence of trauma, middle ear effusions, tonsillar hypertrophy, or exudates. The patient was noted to have bilateral non-tender and mobile anterior cervical lymphadenopathy. An ophthalmologic examination revealed bilateral pulsating pupils and bilateral diplopia of peripheral vision upon straight gaze. Visual acuity using a Snellen eye chart demonstrated 20/70 vision bilaterally. Cardiac auscultation revealed a normal rate and rhythm without murmurs, rubs, or gallops. Their respiratory effort was normal without wheezing, and auscultation was negative for wheezes or rhonchi. There was no abdominal distention or tenderness to palpation, evidence of trauma, or skin rash. Neurological examination revealed dysmetria on finger-to-nose testing, an ataxic gait, positive Romberg, and an inability to perform tandem or tiptoe walking due to unsteadiness. The child otherwise had normal strength, sensation, and intact cranial nerves. Labs from an outside hospital demonstrated mild eosinophilia of 5.2% and an anion gap of 14.9 (Table 1).

**Table 1:** Outside hospital labs. This table details lab values obtained from the outside hospital’s emergency room. BUN = blood urea nitrogen; AST = aspartate transaminase; ALT = alanine transaminase; WBC = white blood cell; RBC = red blood cell; Hgb = hemoglobin; Hct = hematocrit; MCV = mean corpuscular volume; MCH = mean corpuscular hemoglobin; MCHC = mean corpuscular hemoglobin concentration; RDW = red cell distribution width; MPV = mean platelet volume

Lab	Result	Reference range	Lab	Result	Reference range
Sodium	140	136–145 mmol/L	WBC	5.9	5–14.5 K/mm^3^
Potassium	3.9	3.5–5.1 mmol/L	RBC	4.64	4–5.2 × 10^6^/ µ L
Chloride	103	98–107 mmol/L	Hgb	12.9	11.5–15.5 g/dL
Carbon dioxide	26	21–32 mmol/L	Hct	38.4	35–45%
Anion gap	14.9	3.0–15.0 mmol/L	MCV	82.8	76–90 fL
BUN	11	7–18 mg/dL	MCH	27.8	26–30 pg
Creatinine	0.54 (L)	0.55–1.30 mg/dL	MCHC	33.6	32–36 g/dL
BUN/Creatinine ratio	20.4	9.3–24.4	RDW	13.1	11.5–14%
Glucose	96	74–106 mg/dL	Platelet count	201	150–450 K/mm^3^
Calcium	9.4	8.5–10.1 mg/dL	MPV	9.1 (H)	7.8–8.8 fL
Calcium adjusted for albumin	9.1	8.8–10.5 mg/dL	Neutrophil %	36.2	33–76%
Ionized calcium	3.9	3.8–4.8 g/dL	Lymphocyte %	52.0	15–61%
Total bilirubin	0.4	0.2–1.0 mg/dL	Monocyte %	5.9 (H)	0–5%
AST	22	15–37 IU/L	Eosinophil %	5.2 (H)	0–3%
ALT	25	12–78 IU/L	Basophil %	0.5	0–1%
Alkaline phosphatase	202	83–331 IU/L	Absolute neutrophil	2.14 (L)	2.8–6.3 K/mm^3^
Total protein	7.7	6.4–8.2 g/dL	Absolute lymphocyte	3.08 (H)	1.2–2.8 K/mm^3^
Albumin	4.4	3.4–5.0 g/dL	Absolute monocyte	0.35 (L)	0.4–0.9 K/mm^3^
Globulin	3.3	2.8–4.4 g/dL	Absolute eosinophil	0.31 (H)	0–0.2 K/mm^3^
Albumin/Globulin ratio	1.3	1.3-2.8	Absolute basophil	0.03	0–0.1 K/mm^3^

Initial management included consultations with the Poison Control Center and ophthalmology. The patient was admitted to the general pediatrics service for further observation, but no other supportive management was provided. The child’s ataxia and dysmetria steadily improved over the next 10 hours. The child’s vision returned to normal and the pulsating pupils resolved within the same 10-hour time period. Social work was consulted and an outpatient home visit was scheduled as part of an evaluation for child safety given the child’s neurotoxicity after receiving a medication intended for animal use only. The patient was discharged home with complete resolution of all symptoms and with arrangements for an outpatient ophthalmology follow-up.

## Discussion

Our case illustrates the presentation of a nine-year-old with acute-onset ataxia and visual disturbances secondary to the administration of a supratherapeutic dose of veterinary-grade ivermectin. Symptoms completely resolved within 10 hours of admission and the child did not suffer any sequelae from this event. Despite the increase in the incidence of ivermectin toxicity in the United States, there is sparse literature on this topic. Our intention in presenting this case is to aid in ivermectin toxicity identification and hasten the supportive care necessary for treatment.

Ivermectin neurotoxicity is an important consideration in children presenting with unexplained acute neurologic symptoms. Our experience demonstrates potential effects on vision and coordination in a child with previously well-tolerated off-label veterinary ivermectin ingestion. A prior case report illustrated a six-year-old patient who presented with seizures, altered mental status, and hemodynamic collapse requiring intubation following accidental ingestion of 600 mg (30 mg/kg) topical ivermectin, 100 times the intended dose [7]. Our case supports that children may develop neurotoxicity from smaller ivermectin ingestions as well. Although there is no reversal agent available, early diagnosis may minimize otherwise unnecessary evaluation and aid in preparing the medical team for possible neuromuscular collapse. As demonstrated by our patient’s experiences, the neurotoxicity side effect profile of ivermectin is dose-dependent. Ivermectin stimulates glutamate-gated chloride channels on nerve and muscle cells, hyperpolarizing them and preventing action potentials in invertebrates [8]. In mammals, ivermectin potentiates gamma-aminobutyric acid (GABA)-gated currents and reversibly activates the GABA_A_ receptor. Others postulate that ivermectin acts at sites recognized by benzodiazepines on GABA-receptor complexes of chloride channels, which can explain its central nervous system (CNS) depression. Peripherally, ivermectin also potentiates the response of neuron alpha-7 nicotinic acetylcholine receptors (nAChR), which affect the release of glutamate, GABA, norepinephrine, and dopamine [8]. At therapeutic doses (0.1 mg/kg in children and 0.2 mg/kg in adults), multi-drug resistant-1 (MDR1) channels in the blood-brain barrier remove ivermectin, preventing CNS toxicity. However, at supratherapeutic doses, it is theorized that MDR1 channels become saturated and unable to completely remove ivermectin from the brain.

In contrast to the limited existing severe presentations described in the literature, this case depicts a relatively mild case of ivermectin overdose. Our patient did not require any supportive care for respiratory distress or hemodynamic collapse following an overdose of ivermectin. However, it is also important to consider additional adverse effects, including the traumatic nature of a frightening event and subsequent hospitalization, on a young child.

Descriptions of the mechanism of action of ivermectin toxicity and its ability to overwhelm the MDR1 channels in the blood-brain barrier are consistent with our patient’s presentation of ataxia. Ivermectin’s reversible activation of both GABA_A_ channels and alpha-7 nAChR could explain her diplopia due to uncoordinated muscle firing or relaxation. Visual changes could be explained by either ivermectin’s CNS or peripheral effects. As such, our case study provides a salient example of the multi-factorial side effects that follow an ivermectin overdose. This evidence of the physical manifestations of ivermectin side effects strengthens arguments for these aforementioned mechanisms of action and provides an important example of what early high-level ivermectin toxicity can present or provide an archetype of a mild case.

## Conclusions

We present the case of a nine-year-old with neurotoxicity following a 10-fold overdose of oral veterinary ivermectin for COVID-19 prophylaxis. With the increasing off-label use of ivermectin for attempted COVID-19 prophylaxis, our case serves to illustrate the symptoms of ivermectin toxicity in children, which may help identify toxic ingestions. In addition to highlighting the clinical presentation of ivermectin toxicity in children, our case raises important ethical considerations. This child experienced neurotoxicity from the off-label administration by their parents of medication intended for large animals. The involvement of child protective services is critical when a child experiences harm to ensure there is no risk of abuse or further injury. However, the medication was given to the child by parents who intended to help their child based on widely propagated misinformation. Given the substantial increase in inappropriate, potentially harmful, but often well-intended, use of ivermectin for attempted COVID-19 prophylaxis, there is an important need to further explore the ethics of parental intent, autonomy, and beneficence. We hope that this case orients physicians to a usually uncommon occurrence of ivermectin toxicity, provides them with an understanding of the mechanism of these side effects, encourages vigilance for a hemodynamic collapse in these patients, and sparks further discussion regarding the use of veterinary-grade ivermectin as a form of medical neglect.
